# Religion and flourishing among Afro-Trinidadians: the influence of proneness to guilt/shame and forgiveness by God on the relationship between religious commitment and health

**DOI:** 10.3389/fpubh.2024.1443960

**Published:** 2024-12-19

**Authors:** Sebastian Binyamin Skalski-Bednarz, Loren L. Toussaint, Jon R. Webb, Colwick M. Wilson, Everett L. Worthington, David R. Williams, Sandra D. Reid, Janusz Surzykiewicz

**Affiliations:** ^1^Faculty of Philosophy and Education, Catholic University of Eichstätt-Ingolstadt, Eichstätt, Germany; ^2^Institute of Psychology, Humanitas University, Sosnowiec, Poland; ^3^Department of Psychology, Luther College, Decorah, IA, United States; ^4^Department of Community, Family, and Addiction Sciences, Texas Tech University, Lubbock, TX, United States; ^5^School of Social Sciences, University of the Southern Caribbean, St. Joseph, Trinidad and Tobago; ^6^Department of Psychology, Virginia Commonwealth University, Richmond, VA, United States; ^7^Department of Social and Behavioral Sciences, Harvard T.H. Chan School of Public Health, Boston, MA, United States; ^8^Department of African and African American Studies, Harvard University, Cambridge, MA, United States; ^9^School of Medicine, The University of the West Indies (The UWI), Champs Fleurs, Trinidad and Tobago; ^10^Faculty of Education, Cardinal Stefan Wyszyński University in Warsaw, Warsaw, Poland

**Keywords:** forgiveness by god, religious commitment, health, guilt, shame

## Abstract

**Background:**

Religiousness has been consistently linked to positive health outcomes and flourishing, yet the underlying mechanisms are complex and not well-understood. The forgiveness and relational spirituality model offers a framework to explore the moderated mediation among religious commitment, health, and forgiveness by God. Understanding these relationships among university students and community residents in Trinidad and Tobago can provide valuable insights into the role of religiousness in promoting wellbeing.

**Materials and methods:**

This cross-sectional study involved 254 religious Afro-Trinidadians aged 18–78 from Trinidad and Tobago. Participants completed measures assessing religious commitment, proneness to guilt/shame, forgiveness by God, and health outcomes. Modeling using 5,000 bootstrap confidence intervals was used to analyze the hypothesized associations among variables.

**Results:**

A significant positive relationship between religious commitment and health encompassed both physical and mental aspects. Feeling forgiven by God mediated the link between religious commitment and health outcomes (*B* = 0.019; *SE* = 0.007; 95% CI = 0.007, 0.034; *R*^2^ = 0.12). Proneness to guilt or shame moderated the association between religious commitment and forgiveness by God. This relationship was significant primarily among individuals with high levels of proneness to guilt (*B* = 0.075, *SE* = 0.019, *p* < 0.001) and shame (*B* = 0.074, *SE* = 0.018, *p* < 0.001).

**Conclusions:**

This study highlights the importance of religiousness in promoting human flourishing among Afro-Trinidadians residing in Trinidad and Tobago. Religious commitment and forgiveness by God were effective resilience resources that contributed to positive health outcomes. However, the nuanced role of proneness to guilt or shame underscores the need for a deeper understanding of individual differences in emotional responses within religious contexts. Future longitudinal research is warranted to elucidate the dynamic nature of these relationships and inform targeted interventions aimed at enhancing wellbeing in religious communities.

## 1 Background

*Flourishing*, a concept rooted in positive psychology, has garnered significant attention in recent years across diverse academic disciplines including psychology, philosophy, and public health. At its core, flourishing represents a state of optimal wellbeing characterized by the presence of positive emotions, engagement, meaning, positive relationships, and accomplishment (1–4). This holistic perspective on wellbeing transcends the mere absence of illness or distress, emphasizing the importance of thriving and realizing one's full potential in life (5). A vital element of flourishing is that it encompasses good mental and physical health, which are intrinsic to flourishing, rather than merely being outcomes of flourishing. Individuals who experience flourishing are more likely to exhibit higher levels of mental wellbeing, including increased life satisfaction, positive affect, and resilience (6–8). Moreover, flourishing has been associated with aspects of physical health such as better cardiovascular health, lower risk of chronic diseases, and improved immune function (9, 10).

Among minority groups, encompassing both African-Americans and Caribbean Blacks, flourishing can be impeded by racism, characterized as a structured system that categorizes and stratifies social groups into races and systematically devalues, disempowers, and disparately allocates societal opportunities and resources based on racial distinctions (11–14). For example, the pervasive existence of negative cultural representations of individuals of color persists within mainstream American culture, profoundly impacting the emotional wellbeing of these racial minorities. A link between internalized racism and adverse physical health outcomes (i.e., heightened cortisol secretion and metabolic risks) has been demonstrated among Black populations, including African-Americans, Caribbean-born individuals, and Caribbean natives (15–18).

The situation in Trinidad and Tobago presents a unique case where some believe the country's racial and ethnic mixture creates a prejudice-free society (19). However, research by Pierre et al. (20) reveals a different reality, showing that Afro-Trinidadians experience significantly more race-related stress than Indo-Trinidadians and those of mixed ancestry. These disparities arise from both direct personal racism and broader systemic discrimination. Despite its relatively strong economic position within the Americas, Trinidad and Tobago still experiences high poverty rates, and its economic, political, and social structures are influenced by racism. The country, with the largest ethnoracially diverse population in the Anglophone Caribbean, faces challenges in achieving true racial equity (21). Research by Kelly (22), using census data from 1970 to 2011, reveals that while significant White/Black disparities have gradually reduced and some progress has been made in closing the gap between Blacks and East Indians, parity has not yet been achieved. These findings underscore the persistent and complex nature of racial inequity in Trinidad and Tobago.

Due to its potentially health-promoting effects (23), religiousness can serve as a valuable resource in navigating the challenges posed by racial discrimination. Religiousness is a multifaceted construct encompassing both intrinsic dimensions, such as personal beliefs, spiritual experiences, and internalized religiousness, and extrinsic dimensions, including religious practices, behaviors, and community participation (24, 25). These dimensions collectively shape individuals' wellbeing, with intrinsic religiousness fostering a deeper sense of purpose and personal transformation, while extrinsic religiousness provides essential social support and a sense of belonging (26). Longitudinal data consistently highlights a unidirectional effect of religiousness on health outcomes (27, 28). Taylor et al. (29) further observed that religious participation is consistently more prevalent among Black individuals compared to their lighter-skinned and White counterparts, highlighting the significance of exploring religiousness within this demographic. Multiple studies have indicated that religious attendance correlates with heightened subjective wellbeing and reduced psychological distress and mental health disorders overall, including within the Black community (30–32).

It is probable that among Afro-Trinidadians, religion and religious commitment will be a safeguarding element. *Religious commitment* is characterized by adherence to religious values and practices, integration into daily life, and viewing the world through religious schemas (33). This commitment is exemplified by engagement in spiritual practices and reliance on religious beliefs to navigate life's adversities. Therefore, religious commitment is a potential predictor of flourishing within this demographic (34). Understanding the role of religious commitment in fostering flourishing among Black individuals provides valuable insights into resilience and coping strategies amidst racial adversity. Such understanding could contribute to a deeper comprehension of wellbeing in this population (35).

*Forgiveness by God*, also referred to as divine forgiveness, is a core tenet in many theological traditions (36). Frequently introduced in religious texts such as the Bible and the Quran, it is closely associated with divine mercy, yet distinct in its emphasis on absolution of sins and reconciliation with God. For instance, in the Bible, Psalm 51 recounts King David's heartfelt plea for forgiveness after his moral failings, illustrating the depth of divine mercy and the transformative power of being forgiven (Psalm 51:1–19). Similarly, the story of the prophet Jonah in the Book of Jonah, who finds redemption after repenting during his time in the belly of a great fish, underscores the importance of divine forgiveness in restoring one's relationship with God (Jonah 2:1–10). For the religiously committed, this concept is often pivotal in their spiritual journey, contributing to spiritual growth and deeper understanding (37). Defined as the act of liberating individuals from transgressions or sins by God, divine forgiveness offers a profound sense of redemption, reconciliation, and spiritual liberation (38–41). Divine forgiveness has been identified as a powerful mechanism through which individuals experience emotional healing and restoration. Numerous studies underscore the importance of feeling forgiveness by God in promoting psychological wellbeing and resilience, providing believers with relief from guilt and shame, and (thereby) buffering them against the detrimental effects of stress and adversity (40, 42). Moreover, belief in divine forgiveness has been associated with increased levels of optimism, hope, and coping strategies, which in turn contribute to physical and mental health (43). Toussaint and Williams (39) discovered that among a representative sample of adults in the United States, feeling forgiven by God correlated with a reduced likelihood of depression among women. Similarly, Lawler-Row (44) identified notable connections between feeling forgiven by God and depressive symptoms in older adults. Furthermore, among college students, a feeling of divine forgiveness was associated with fewer depressive symptoms both at baseline and 3 years later (45, 46). Additionally, Krause and Ellison (47) provided empirical evidence indicating that the sensation of being forgiven by God fully mediated the relationship between indicators of religiousness and depression. They proposed a theological interconnectedness between religion and forgiveness, suggesting that the two factors exert similar effects on health. Consequently, they proposed that forgiveness may represent an important pathway through which the association between religion and health operates. This proposition aligns with the earlier findings of McCullough and Worthington (42), who conducted a comprehensive study utilizing data from three forgiveness studies, two newly conducted and one derived from archival data. They asserted that “If relationships between forgiveness and various measures of health and wellbeing turn out to be robust and nonspurious, we will have all the more reason to place forgiveness squarely in the middle of the religion-health interface” (p. 116).

Supplementing empirical findings, the forgiveness and relational spirituality model (48, 49) offers a comprehensive framework for examining the potential mediating role of feeling forgiven by God in the relationship between religious commitment and health. Within this model, spirituality is understood as a central dimension of human existence that shapes meaning, purpose, and connection with a transcendent, though not necessarily theistic, reality. Rooted in the integration of forgiveness and spirituality within the context of inter- and intra-personal relationships, this model highlights their reciprocal influence. It posits that forgiveness is intricately intertwined with an individual's spiritual beliefs and relational experiences (50). According to the model, spiritual beliefs and practices play a pivotal role in facilitating forgiveness by infusing conflicts with meaning, purpose, and guidance, thereby fostering qualities such as empathy, compassion, and a yearning for reconciliation (51). Also, forgiveness can deepen one's spiritual connection by nurturing (and drawing on) humility, grace, and unconditional love (52). Within this theoretical context, one's sense of experiencing forgiveness by God could serve as a mediating variable to elucidate the impact of religious commitment on flourishing.

Although prior research has established a foundation for exploring the intricate relationship between religious commitment and the pursuit of forgiveness from God (50, 53), achieving a comprehensive understanding of experiencing divine forgiveness requires investigating individual differences that may evoke it. Within the realm of psychological inquiry, an intriguing avenue emerges: examining the potential buffering role of proneness to guilt and shame. Historically, shame and guilt were frequently conflated and treated interchangeably in conceptual terms (54). However, Lewis (55) clarified that *guilt* pertains to a negative emotional response following a specific action, while *shame* encompasses a broader negative perception of oneself.

Guilt, often associated with negative arousal, serves a significant function in maintaining and repairing relationships (56). Baumeister et al. (57) demonstrated its role as a motivator for seeking forgiveness, as those feeling guilty about causing offense were more likely to apologize. Similarly, guilt has been linked to repairing relationships (58). Riek's (59) study indicated guilt as a predictor of seeking forgiveness, and a longitudinal study by Riek et al. (60) further supported the role of guilt in determining forgiveness-seeking behavior. In contrast, Jordan et al. (61) revealed that both trait-based and state-based guilt prompted transgressors to extend forgiveness to unrelated third-party transgressors, driven by an increased sense of identification with these individuals. This identification may extend to the relationship between individuals and God, to whom personal offenses and insults were directed, particularly in the context of divine forgiveness.

Kim (62) explored the relationship with God, highlighting that guilt and shame arise from an awareness of one's shortcomings when measured against divine standards. While shame reflects a negative evaluation of the self, guilt is tied to specific behaviors and fosters a sense of responsibility to reconcile with God. Both emotions may influence personal worth and behavior, playing roles in developing responsibility and aligning moral values with divine expectations.

Research on shame within the context of forgiveness remains relatively limited. While many studies underscore the adverse effects of shame (63, 64), Murray and Ciarrocchi (65) proposed that shame also has a constructive aspect, serving as a reminder of humanity and fostering stronger bonds. Within the framework of transcendence, these stronger connections may reinforce belief in God's unrivaled dignity and everlasting love through granting forgiveness.

These findings suggest promising avenues for further investigation within the realm of religious commitment. Individuals with heightened religious devotion may be inclined to embark on a deeper journey toward experiencing forgiveness from a divine entity and reconciling their transgressions with the entity. This pursuit is likely influenced by emotional responses of guilt and shame, which foster identification with God. This heightened identification could result in deeper engagement in the process of seeking forgiveness from and contentment with God, and coming to a sense of contentment with oneself. In line with this, individuals experiencing guilt and shame are inclined to perceive the self and God as sharing a common goal, based on the act of rescue from the power of sin. Moreover, strong identification has the potential to lead to an emotional merging of oneself, prompting individuals to view the target of identification favorably (66).

Regarding divine forgiveness's direct response to individual transgressions, Hall and Fincham (67) and Skalski-Bednarz (68) highlight that perceived forgiveness by God is associated with a subsequent increase in self-forgiveness. The proposed moderating influence of guilt and shame on the relationship between religious commitment and health, facilitated by forgiveness by God, can be understood through *stress-and-coping models of forgiveness* (69, 70), particularly focusing on self-forgiveness (71, 72), rooted in Lazarus and Folkman's (73) transactional theory of stress and coping. These frameworks indirectly suggest that self-condemnation—a stressful state characterized by negative emotions—may motivate individuals to seek and receive divine forgiveness as a coping mechanism, which, in turn, could facilitate self-forgiveness. Furthermore, the stress-and-coping models of forgiveness suggest that aspects of self-condemnation such as shame and guilt may serve as important moderators of associations of religiousness and flourishing. This is because experiences of shame and guilt provide a need for divine forgiveness for religious individuals, and as such, catalyze coping mechanisms such as divine forgiveness which ties religiousness to flourishing.

In the current study, we aim to explore the relationship between religious commitment and flourishing among Afro-Trinidadians. Building upon the existing literature, we hypothesize that proneness to guilt and shame moderates the mediation of forgiveness by God in the positive relationship between religious commitment and health. Specifically, we predict that the mediating effect of God's forgiveness will be significant when individuals exhibit high levels of proneness to shame or guilt. These findings have significant implications for understanding the intricate interplay between religious beliefs, emotional experiences, and health outcomes among Afro-Trinidadian individuals residing in Trinidad and Tobago. They may also inform interventions aimed at promoting psychological wellbeing and spiritual growth among this population.

## 2 Materials and methods

Ethics approval for this study was obtained from the Institutional Review Board of the University of the Southern Caribbean (USC-RE-REC-002-2023-0274). Informed consent was provided by all participants after receiving detailed information about the study procedure. The information conveyed included the characteristics, applications, advantages, and potential drawbacks of the research, and participation was entirely voluntary.

### 2.1 Participants and procedure

This dataset was derived from a broader survey on flourishing in Trinidad and Tobago conducted in 2023 using the online platform Qualtrics. Participation was restricted to adult university students and community residents residing in Trinidad and Tobago, with open religious affiliation and no other prerequisites. Participants were recruited from community residents and university student groups and also via social media, including Facebook and X. Based on the premise that Afro-Trinidadians may experience more stress related to racism (20, 22) and that religion is a more frequent coping strategy among Black people compared to other racial groups (32), the sample for this analysis was narrowed to Afro-Trinidadian adults residing in Trinidad and Tobago, regardless of their religious affiliation. After applying these criteria, 31 participants were excluded. The final sample consisted of 254 individuals aged 18–78 (*M*_age_ = 29.5, *SD* = 13.3), where 59.8% were female. Participants exhibited diversity in terms of educational background (26% middle school, 27% high school, 57% college) and marital status (65% were partnered). The study procedure involved completing questionnaires on religious commitment, proneness to shame and guilt, divine forgiveness, and health, with an estimated completion time of ~15 min for this selected subset of measures.

### 2.2. Measures

Religious commitment was assessed using the *Religious Commitment Inventory-10* (RCI-10), a ten-item scale developed by Worthington et al. (33). This instrument evaluates both interpersonal and intrapersonal dimensions of religious commitment utilizing a five-level response format (1 = not at all true of me, 5 = totally true of me). Total scores, reflecting the extent of commitment to religious beliefs, values, and practices, and the degree of integration of these elements into various aspects of life, were computed by summing responses. The RCI-10 demonstrated robust psychometric properties, with 3.-week test-retest coefficients ranging from 0.84 to 0.87 (33). Furthermore, RCI-10 scores exhibited positive correlations with self-reported attendance at religious events, self-ratings of spirituality, and reported spiritual intensity, as evidenced by Worthington et al. (33). In our study, internal consistency reliability was estimated at α = 0.93, aligning with the reported range of 0.88–0.98 across diverse samples by Worthington et al. (33). Sample items included statements like “My religious beliefs underlie my whole approach to life.” and “I enjoy working in the activities of my religious organization.” Response options ranged from 1 = *Not at all true of me* to 5 = *Totally true of me*.

The evaluation of shame and guilt utilized the *Personal Feelings Questionnaire 2 Brief* (PFQ-2 Brief), developed by Rice et al. (74). This succinct seven-item scale, adapted from the PFQ-2 (75), aims to gauge the frequency of individuals' experiences with shame and guilt. Respondents are prompted to consider how often they encounter affective states associated with these constructs. The scale employs a five-level response format (1 = *never experience*, 5 = *constantly or almost constantly experience*). Participants rated three items related to guilt and four items related to shame. The resulting scores on these scales capture trait levels of proneness to shame and guilt. Despite its brevity, the seven-item scale demonstrated a robust model fit, strong convergent validity, and superior psychometric properties compared to the original PFQ-2. In our sample, the Cronbach alphas for the guilt-proneness and shame-proneness scales were 0.79 and 0.82, respectively. Sample items included assessing the commonality of feelings such as “feeling humiliated” (shame) and “mild guilt” (guilt).

Perception of divine forgiveness was assessed using the *Divine Forgiveness Scale* (DFS), developed by Fincham and May (76). The total score, reflecting the perceived extent of God's forgiveness, was computed as a sum of four items rated using five-point response options, with higher scores indicating a greater sense of perceived divine forgiveness. In our sample, the estimated internal consistency reliability of the DFS was assessed, yielding a Cronbach's alpha of 0.79. Sample items included statements such as “How often have you felt that God forgives you?” (1 = *never*, 5 = *many times*) and “I am certain that God forgives me when I seek his forgiveness” (1 = *strongly disagree*, 5 = *strongly agree*).

Perceived health was evaluated using the *PROMIS Global Health* scale (77), comprising two subscales, physical health and psychological wellbeing, each consisting of two items. This scale was chosen to efficiently capture key dimensions of flourishing (7) while minimizing the time required for participant responses. Respondents rated each statement using five-point response options (1 = *excellent*, 5 = *poor*). Total scores ranged from 4 to 20. To enhance clarity in interpretation, we reversed the coding of this scale, ensuring that a higher score indicates greater presence of health. In this study, we employed a second-order health factor encompassing both mental and physical dimensions. This factor was derived from a principal components factor analysis with oblimin rotation, explaining 59.15% of the variance; all items loaded above 0.65 on the factor. Additionally, the estimated internal consistency reliability of this second-order incorporated factor was assessed, resulting in a Cronbach's alpha of 0.78. Sample items included questions such as, “In general, how would you rate your physical health?” and “In general, how would you rate your mental health, including your mood and your ability to think?”

Finally, we utilized two widely used single-item questions to assess general religiousness (“To what extent do you consider yourself a religious person?”) and spirituality (“To what extent do you consider yourself a spiritual person?”) Participants responded to both questions using five-point response options (1 = *not at all*, 5 = *extremely*). Furthermore, the first question was employed to exclude participants who did not meet the recruiting criterion of being a religious person; eight individuals were exempted based on their response indicating “*not at all*.”

### 2.3. Statistical data analysis

The analyses were conducted using IBM SPSS Statistics 29 software and the PROCESS 4 (78) plugin for moderated mediation effects analysis (Model 7). The Kolmogorov–Smirnov test assessed the normality assumption; the homoscedasticity of variance was checked using Levene's test. Parametric tests were applied due to the normal distribution of the data. Evaluation of associations between variables employed Pearson's *r*. In the context of moderated mediation analysis, bias-corrected bootstrapped 95% confidence intervals for indirect effects were obtained from 5,000 bootstrapped samples. To illustrate moderation effects effectively, procedures outlined by Aiken et al. (79) were adhered to. Conditional indirect effects were examined at one standard deviation (*SD*) above the mean, at the mean, and at one *SD* below the mean for the moderator variable of interest. This examination aimed to ascertain whether the slopes of regression equations for high and low values of the interaction differed significantly from zero. The effect size was gauged using *R*^2^. The significance level was established at *p* ≤ 0.05.

## 3 Results

Religious commitment exhibited a moderate positive correlation with divine forgiveness, a small positive association with health, and small negative links with proneness to guilt and shame. Divine forgiveness displayed small correlations with low proneness to guilt and shame and greater health, while proneness to guilt and shame were strongly intercorrelated and exhibited moderate negative links with health. Descriptive statistics and bivariate correlation coefficients are presented in Table 1, which also includes sociodemographic data such as age, sex, religiousness (note that due to the exclusion of non-religious individuals, the actual response scale for this question ranged from 2 to 5; see Section 2.2.), and spirituality. Before conducting the moderated mediation analysis, a collinearity test was performed to assess the correlation between potential predictors. The variance inflation factor (VIF) ranged between 1.11 and 1.16, indicating the absence of collinearity between independent variables.

**Table 1:** Means and correlations among Afro-Trinidadians (*N* = 254).

	***M* (*SD*)**	**1**.	**2**.	**3**.	**4**.	**5**.
1. Religious commitment	33.3 (11.4)	–				
2. Divine forgiveness	14.7 (2.3)	0.31^***^				
3. Guilt proneness	9.3 (2.4)	−0.26^***^	−0.24^***^	–		
4. Shame proneness	10.1 (3.1)	−0.28^***^	−0.26^***^	0.74^***^	–	
5. Health	14.2 (2.8)	0.27^***^	0.29^***^	−0.42^***^	−0.44^***^	–
Age	39.2 (15)	0.07	0.10	−0.09	−0.10	0.03
Sex (1 = male, 2 = female)	1.6 (0.4)	−0.06	−0.04	0.09	0.08	0.05
Religiousness	3.5 (1)	0.77^***^	0.19^**^	−0.18^**^	−0.19^**^	0.22^***^
Spirituality	3.7 (1)	0.69^***^	0.27^***^	−0.21^***^	−0.22^***^	0.30^***^

^**^p ≤ 0.01.

^***^p ≤ 0.001.

### 3.1. Mediation analysis

**Figure 1:**
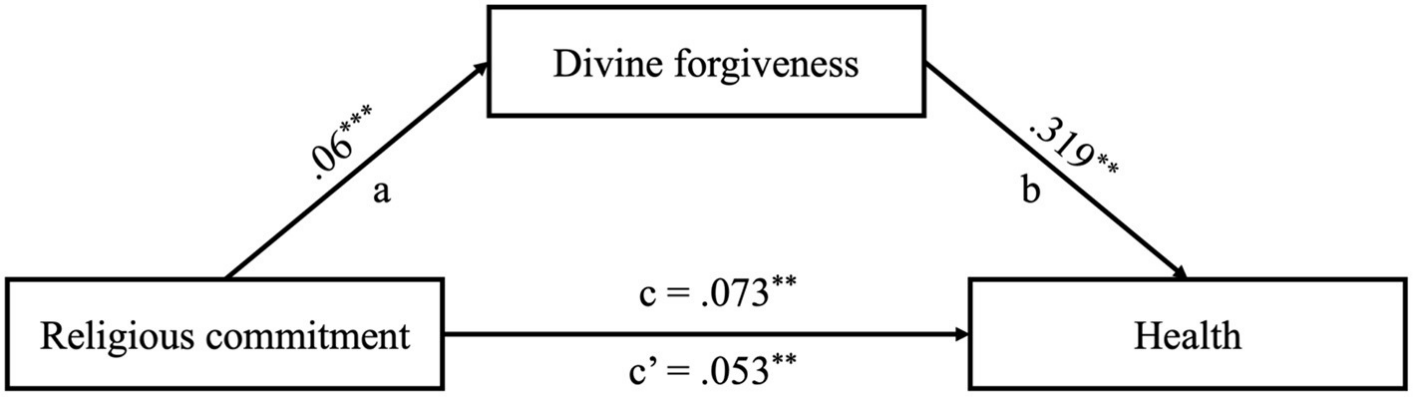
Results of the mediational model among Afro-Trinidadians (*N* = 254). ***p ≤ 0.001, **p ≤ 0.01. Unstandardized coefficients are presented.

The bootstrap sampling analysis demonstrated a statistically significant indirect effect of divine forgiveness in the relationship between religious commitment and health (indirect effect; *B* = 0.019; *SE* = 0.007; *95% CI* = 0.007, 0.034; *R*^2^ = 0.12). The analysis indicated a positive and statistically significant direct relationship between religious commitment and health (total effect; *B* = 0.073; *SE* = 0.020; *95% CI* = 0.034, 0.110; *R*^2^ = 0.07). Upon the inclusion of the potential mediator, divine forgiveness, the relationship between religious commitment and health decreased by 29% but remained statistically significant (direct effect; *B* = 0.053; *SE* = 0.020; *95% CI* = 0.014, 0.093). Furthermore, religious commitment was identified as a positive predictor of divine forgiveness (*B* = 0.061; *SE* = 0.014; *95% CI* = 0.033, 0.087; *R*^2^ = 0.10). A visualization of the potential mediation model is presented in Figure 1. For true mediation, a longitudinal design is necessary in subsequent research.

### 3.2. Moderated mediation analyses

**Figure 2:**
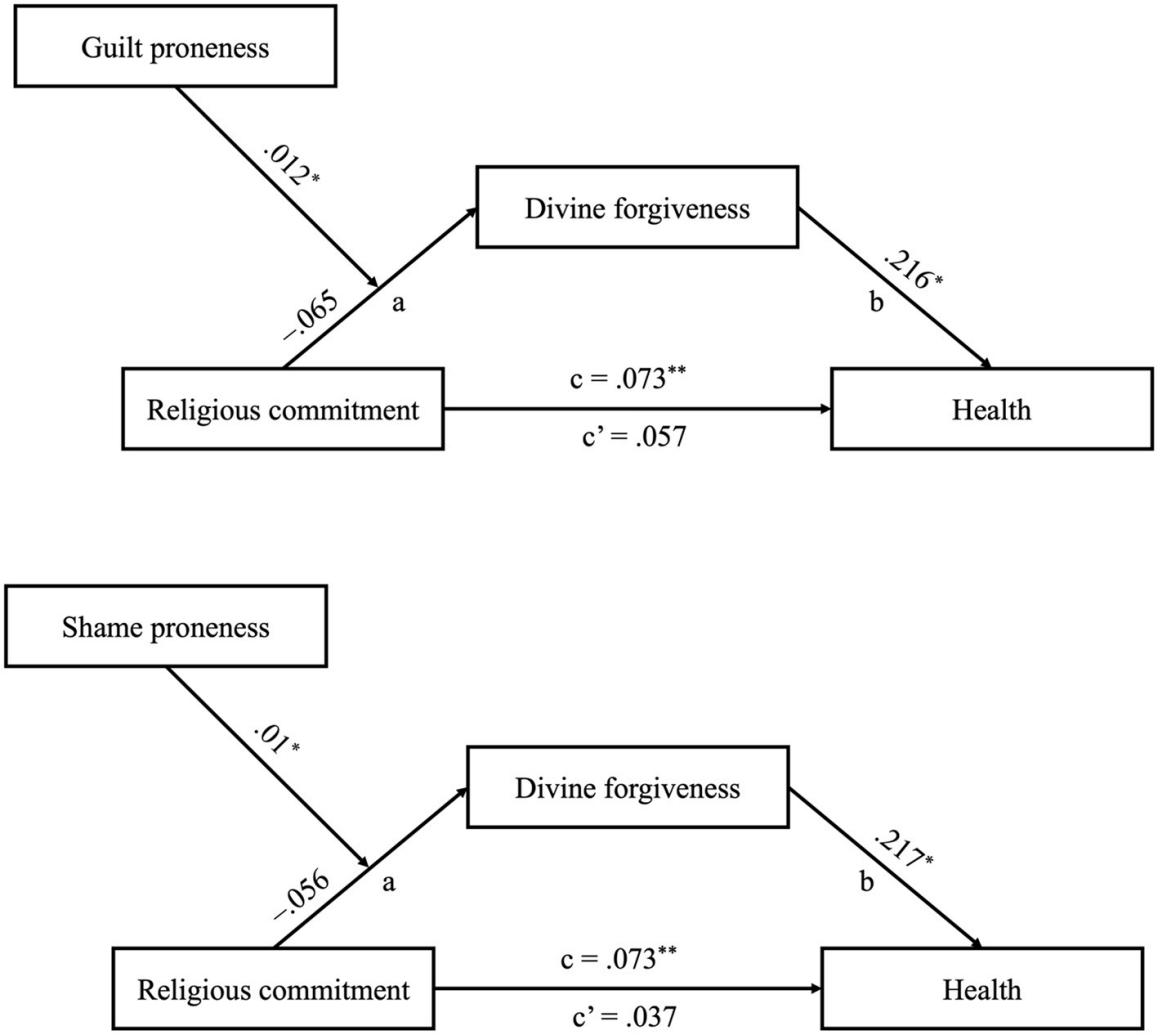
Results of the moderated mediational model among Afro-Trinidadians (*N* = 254). **p ≤ 0.01, *p ≤ 0.05. Unstandardized coefficients are presented.

In a subsequent step, moderated mediation analysis was conducted to examine the relationships among proneness to guilt and shame, religious commitment, divine forgiveness, and health. Given the earlier reporting of the potential mediation finding from the mediational analysis, this section is dedicated to direct presentation of potential interaction effects between the variables within the hypothesized regression models. As outlined in Table 2, the findings revealed a significant interaction effect between religious commitment and proneness to shame on the likelihood of feeling divine forgiveness (*p* = 0.023). Additionally, a significant interaction was observed between religious commitment and guilt-proneness regarding divine forgiveness (*p* = 0.025). The final potential moderated mediation models are displayed in Figure 2. Notably, alternative models assessing proneness to shame and guilt as potential moderators in the relationships between religious commitment and health, and between divine forgiveness and health, did not yield significant effects (*p* > 0.05).

**Table 2:** Interaction effects between religious commitment and proneness to shame and guilt on divine forgiveness among Afro-Trinidadians (*N* = 254).

	***B***	***SE***	***t***
Religious commitment	−0.065	0.051	−1.267
Guilt proneness	−0.531	0.185	−2.863^**^
Religious commitment x Guilt	0.012	0.005	2.304^*^
*R* ^2^	0.14		
Religious commitment	0.049	0.015	3.328^***^
Guilt proneness	−0.135	0.07	−1.921
*R* ^2^	0.11		
Religious commitment	−0.055	0.045	−1.221
Shame proneness	−0.438	0.149	−2.926^**^
Religious commitment × Shame	0.01	0.004	2.352^*^
*R* ^2^	0.14		
Religious commitment	0.046	0.015	3.163^**^
Shame proneness	−0.109	0.054	−2.032^*^
*R* ^2^	0.11		

^*^p ≤ 0.05.

^**^p ≤ 0.01.

^***^p ≤ 0.001.

The links between religious commitment and divine forgiveness were scrutinized through simple main effects analyses at 1 *SD* above and below the means of guilt and shame. At the higher level (mean +1 *SD*) of the guilt-proneness or shame-proneness dimension, the main effects of religious commitment on divine forgiveness were found to be significant. A higher level of religious commitment was positively correlated with greater divine forgiveness (*B* = 0.075, *SE* = 0.019, *p* < 0.001 for guilt-proneness; *B* = 0.074, *SE* = 0.018, *p* < 0.001 for shame-proneness). In contrast, religious commitment did not exhibit a statistically significant association with levels of divine forgiveness at lower levels (mean −1 *SD*) of guilt-proneness or shame-proneness (*p* > 0.05).

To evaluate the positive moderation effects of the focal predictor on the potential mediator across different levels of the moderators, we employed the bootstrap method for analyses. The indirect effects were scrutinized at three levels of proneness to guilt or shame (1 *SD* above the mean, at the mean, and 1 *SD* below the mean) using 95% confidence intervals. As outlined in Table 3, the impact of religious commitment on divine forgiveness decreased across the range of the moderators and eventually reached statistical insignificance at 1 *SD* below the mean of the moderators.

**Table 3:** Conditional effects of religious commitment on divine forgiveness across three levels of shame and guilt among Afro-Trinidadians (*N* = 254).

	**Indirect effect**	***SE***	**LL 95% *CI***	**UL 95% *CI***
**Guilt**				
1 SD below the mean	0.021	0.019	−0.016	0.058
Mean	0.045	0.015	0.016	0.074
1 SD above the mean	0.076	0.019	0.039	0.113
**Shame**				
1 SD below the mean	0.015	0.020	−0.024	0.053
Mean	0.045	0.014	0.016	0.073
1 SD above the mean	0.075	0.019	0.037	0.112

Additionally, we explored the conditional indirect effects of religious commitment on health through divine forgiveness across the same levels of shame-proneness and guilt-proneness among Afro-Trinidadians. These analyses, detailed in Table 4, reveal that the indirect effects were insignificant at 1 *SD* below the mean of the moderators.

**Table 4:** Conditional indirect effects of religious commitment on health through divine forgiveness across three levels of shame and guilt among Afro-Trinidadians (*N* = 254).

	**Indirect effect**	***SE***	**LL 95% CI**	**UL 95% CI**
**Guilt**				
1 SD below the mean	0.005	0.005	−0.003	0.015
Mean	0.01	0.005	0.001	0.021
1 SD above the mean	0.016	0.008	0.003	0.034
**Shame**				
1 SD below the mean	0.004	0.006	−0.005	0.014
Mean	0.011	0.006	0.001	0.021
1 SD above the mean	0.015	0.007	0.002	0.035

### 3.3 Summary of analyses

The results of the analyses reveal that religious commitment is positively associated with flourishing, measured as mental wellbeing and physical health. This relationship is partially mediated by the perception of divine forgiveness, which plays a significant role in explaining how religious commitment contributes to better health outcomes. Furthermore, the association between religious commitment and divine forgiveness is statistically significant, and the mediation effect occurs only when guilt proneness or shame proneness levels are moderate or high. When guilt proneness or shame proneness levels are low, neither the association nor the mediation effect is observed.

## 4 Discussion

Experiences related to racism continue to present significant challenges to the wellbeing of Caribbean Blacks (11, 13, 20). Building upon previous research suggesting higher religiousness among Black individuals (29), engagement in spiritual practices has been recognized as a vital resource that fosters flourishing within this demographic (34). The current study, conducted within the Trinidad and Tobago community, extends these findings by elucidating the potentially mediating role of divine forgiveness in the relationship between religious commitment and overall health. Furthermore, consistent with our hypothesis of moderated mediation, we discovered that the positive health impact of feeling divine forgiveness depends on individuals' levels of proneness to guilt and shame. This highlights the intricate interplay between religious involvement, forgiveness, and improved health among Trinidadians of African descent, offering insights into potential pathways for enhancing resilience within this specific population.

The findings of this study enhance our understanding of the relationship between religiousness and wellbeing among Afro-Trinidadians. Our results are consistent with previous research (22–24), which has evidenced positive correlations between religious commitment and both improved subjective wellbeing and reduced psychological distress and mental health disorders within the Black community. Moreover, our study extends this knowledge by revealing that religious commitment also is related to better overall mental and physical health among Afro-Trinidadians. For example, these findings are consistent with the research conducted by Holt et al. (80) among Black Americans, which demonstrated a connection between religious behaviors and increased consumption of fruits and vegetables, reduced alcohol intake, fewer instances of binge drinking, and lower smoking rates, even after adjusting for coping-related factors. In light of Holt et al.'s (80) results, it appears that the protective effect of religious commitment on physical health may stem from religious teachings and sanctions that discourage unhealthy behavior.

### 4.1. Mediating role of divine forgiveness

In addition to the role of religious teachings in promoting pro-healthy behaviors, our study suggests alternative pathways through which religious commitment is correlated with health outcomes among Caribbean Black individuals, necessitating further investigation in future research. Specifically, we found that feeling forgiven by God can act as a mediator in this relationship. This indicates that individuals' perception of divine forgiveness serves as a conduit through which religious commitment may enhance health, supporting the hypothesis proposing divine forgiveness as a mechanism in the religion-health relationship (42). Furthermore, our findings resonate with Lawler-Row's (44) study among older Americans which revealed that feeling forgiven by God fully mediated the associations between religious attendance, prayer frequency, belief in a vigilant God, and successful aging. Thus, the positive life adjustment observed among individuals engaged in religious practices may arise from their sense of being in a relationship with and accepted by their concept of God. This finding aligns with the research conducted by Skalski et al. (81), who examined individuals experiencing disappointment with God during wartime, which was linked to lower levels of dispositional forgiveness. The authors suggested that faith-mature individuals, who can maintain their commitment despite disappointment with God, are more likely to experience improved psychological and physical wellbeing.

### 4.2. Moderation by guilt and shame proneness

Our findings contribute to a framework delineating the process of feeling forgiveness from God, revealing that proneness to guilt or shame moderates the relationship between religious commitment and feeling forgiven by God among Afro-Trinidadians. This suggests that individuals with higher levels of proneness to guilt or shame may be more inclined to embrace the concept of divine forgiveness as a means of alleviating psychological distress and achieving better overall health. These results underscore the importance of considering individual differences in emotional reactions when assessing the influence of religious commitment on health outcomes and offer theoretical insights into prevailing interpretations of divine forgiveness. While forgiveness by God is traditionally understood as absolution from wrongdoing by God (38), our findings suggest that this type of forgiveness requires individuals to acknowledge their personal transgressions, accompanied by feelings of self-condemnation such as guilt or shame. This observation aligns with certain therapeutic strategies that suggest confronting challenging truths early in the process as essential to healing and attaining forgiveness (82, 83). Additionally, our findings resonate with previous research by Riek (59, 60), indicating that factors such as the severity of the offense, relationship intimacy, and rumination influence the propensity to interpersonal forgiveness, many of these relationships being influenced by feelings of guilt, highlighting the role of negative emotions in motivating the desire for forgiveness. Finally, in line with our expectations, guilt and shame played similar roles in regulating positive health effects among Afro-Trinidadians. While existing research often highlights negative or negligible links between shame and interpersonal forgiveness (63, 64), our approach, inspired by Murray and Ciarrocchi (65), suggests a beneficial function for shame and guilt in the realm of divine forgiveness. We propose that shame and guilt, when perceived constructively, act as a reminder of our human frailty. This perspective can deepen one's connection with the divine and enhance their appreciation for God's mercy. Consequently, this realization may motivate individuals to seek reconciliation and pardon from a divine entity like God, aiming to mitigate feelings of shame and guilt and regain moral wholeness, including health.

### 4.3. Practical implications

One practical implication of this study is the importance of addressing feelings of guilt and shame in religious contexts, particularly among Afro-Trinidadians. Given that higher levels of proneness to guilt or shame were associated with a greater inclination to feel forgiveness from God, religious leaders and practitioners can focus on promoting a supportive environment where individuals feel comfortable acknowledging their transgressions and feeling divine forgiveness. This may involve incorporating teachings and practices that encourage self-reflection, self-compassion, and acceptance of imperfections within religious communities. Additionally, mental health professionals working with individuals from these communities can integrate spirituality into therapeutic interventions, recognizing the potential role of divine forgiveness in alleviating psychological distress and promoting overall wellbeing. One example of such an intervention is the *Moving Forward* self-forgiveness intervention (83), which combines principles of religious teachings with evidence-based therapeutic techniques. This intervention typically involves guided exercises and discussions aimed at helping an individual understand self-forgiveness from a Christian perspective, process feelings of guilt and shame, and cultivate a sense of compassion toward oneself and others. By incorporating Moving Forward or similar interventions into religious and therapeutic settings, individuals may be better equipped to navigate challenges, strengthen their spiritual connections, and experience greater emotional and physical health benefits.

### 4.4. Limitations and directions for future research

While this study offers valuable insights, it is essential to acknowledge several limitations. Firstly, the cross-sectional design employed in this study limits our ability to establish causal relationships or explore the dynamic interplay among variables over time. Utilizing a longitudinal cross-lagged panel design in future research would provide a more nuanced understanding of the intricate associations between religious commitment, proneness to guilt or shame, forgiveness by God, and health outcomes. Furthermore, the study focused on a specific population of Afro-Caribbeans in Trinidad and Tobago, which may restrict the generalizability of the findings to other cultural or geographical contexts. Additionally, the study did not account for experiences of racism, which remains a significant barrier to the flourishing of Black individuals. Although it is assumed that the intensity of racism and discrimination may be lower in the home country compared to migration scenarios in regions like North America, future research should consider incorporating measures of experience of racism to understand better its impact on the variables under study. Moreover, this study used two single-item questions to separately assess general religiousness and general spirituality, a common method (84). While Dollinger and Malmquist (85) suggest these can have adequate validity and reliability, they can still lead to variability in construct interpretation. These variables were secondary, with the primary focus on religious commitment and divine forgiveness. Finally, since all participants identified as believers, the study did not control for specific religious affiliations. Different religious beliefs may offer distinct interpretations of divine forgiveness, which could influence the meaning and effects of this construct. Given that the sample likely reflects a predominantly Christian perspective, as Trinidad and Tobago has a substantial Christian population (86), future studies could diversify the sample and account for varying religious affiliations to explore potential differences in perceptions of divine forgiveness. Measuring religious participation in future studies would also be valuable, as previously mentioned, Taylor et al. (29) highlight that Black individuals may more frequently engage in practices compared to lighter-skinned and White counterparts. Addressing these limitations in future research endeavors will enhance the comprehensiveness and applicability of the findings to understand the complex relationship between religion, guilt/shame, forgiveness, and health outcomes.

## 5 Conclusions

In summary, this study sheds light on the intricate relationships among religious commitment, proneness to guilt or shame, forgiveness by God, and health outcomes within the Black Caribbean community in Trinidad and Tobago. Our findings underscore the significant role of proneness to guilt and shame in moderating the connection between religious commitment and feeling forgiven by God, particularly among this specific population. Individuals with higher levels of proneness to guilt or shame tend to feel divine forgiveness more readily, aiming to alleviate psychological distress and improve physical health. However, it is crucial to acknowledge several limitations, including the study's cross-sectional design. Additionally, longitudinal research is needed to explore the dynamics between religious commitment, divine forgiveness, and wellbeing. Both religious commitment and forgiveness by God offer avenues to develop and implement interventions to promote flourishing among Afro-Trinidadians. The identification of forgiveness by God as a mediator between religious commitment and health suggests the potential combined effect of intervening in these two aspects to enhance health and wellbeing, while the moderating effect emphasizes the importance of recognizing self-condemnation in the healing journey.

## Data Availability

The raw data supporting the conclusions of this article will be made available by the authors, without undue reservation.
